# Repelling Fruit Flies with Essential Oils and Their Components: the Peach Fruit Fly *Bactrocera zonata*

**DOI:** 10.1007/s10886-025-01628-9

**Published:** 2025-08-09

**Authors:** Anat Levi-Zada, Sara Steiner, Daniela Fefer, John A. Byers

**Affiliations:** 1 Institute of Plant Protection, Department of Entomology-Chemistry Unit, Agricultural Research Organization, Volcani Institute, Rishon LeZion, Israel; 2 Semiochemical Solutions, Beer Yaakov, 7030476 Israel

**Keywords:** Insect repellents, Trap shutdown, Semiochemicals, Tephritid fruit flies, Field testing, Push-pull, Pest management

## Abstract

The peach fruit fly (PFF) *Bactrocera zonata* (Diptera: Tephritidae) is a major agricultural pest attacking at least 55 plants of economic importance. Previous studies have identified unique compounds in each sex, but none of these appear attractive to either sex. However, it is well known that methyl eugenol (ME) found in nectaries of many plants is strongly attractive to males, while food baits are weakly attractive to both sexes. The present study was designed to find repellent substances for the PFF to implement “push-pull” methods as part of efforts to control the pest and protect orchards. The push-pull method requires an attractant, such as ME, for the fly, and a repellent, which has yet to be discovered. A substance is repellent if it can reduce the attraction of insects to a trap with an attractant as compared to a trap with an attractant alone. The reduction is measured as “trap shutdown”, with 100% shutdown being complete repulsion. After systematically screening 82 commercial essential oils (EOs) presented in combinations in field experiments, we found that two EOs, Yarrow and Ylang-ylang, caused over 95% trap shutdown for PFF males and females at different timings and in different orchards (i.e., citrus and mango). We also tested the components of these two EOs and found that artemisia ketone, a component of Yarrow EO, causes a significant trap shutdown of 98% for males and 92% for females. Each component of Ylang-ylang EO tested in the field did not give a sufficient shutdown, suggesting possible synergism among components. However, this EO is inexpensive and thus can be applied wholly in a suitable formulation. We believe that our findings could be developed for the push-pull of other fruit flies known to be attracted to ME.

**Supplementary Information:**

The online version contains supplementary material available at 10.1007/s10886-025-01628-9.

## Introduction

There are over 650 described species of fruit flies of the *Bactrocera* genus of which at least 50 species are important pests of agriculture (Vargas et al. 2015). The females of many fruit fly species cause damage by laying eggs in the skin of vegetables and fruits, where the larva develops and turns the tissues soft and mushy, disqualifying the fruit or vegetable for marketing. Besides the direct damage to fresh produce, the significant economic importance of these fruit flies is also due to their status as quarantine pests in many countries, which prohibit imports from countries where these pests exist.

The peach fruit fly (PFF), *Bactrocera zonata* (Saunders), is damaging to over 50 economically important plants, and the literature defines the species as one of the most serious pests in agriculture (Azza et al. 2014; Vargas et al. 2015). In the past, we attempted to characterize and identify the pheromones of both male and female PFF individuals. We identified volatile substances unique to males and females that are released in a circadian manner by both sexes (Levi-Zada et al. 2020). However, as with most fruit fly pheromones of a similar nature, we had difficulties proving behavioral activity other than the specific volatiles stimulating the PFF antennae.

PFF males and other males of *Bactrocera* species are attracted to the volatile methyl eugenol (ME), which is found in the flowers and nectar of many plants in the fruit flies’ natural habitat (Tan and Nishida 2012). Thus, ME can be used in integrated pest management (IPM) to monitor the level and distribution of the pest population for more effective control, utilizing a combination of pesticides, the dispersal of sterilized males (SIT), and mass trapping of males (Vargas et al. 2012, 2015). However, pest control of *Bactrocera* species using ME is not sufficient because only males are targeted, while females, which cause most of the damage, are not attracted by ME. In addition, as with many species of fruit flies, it is possible to attract females and males of PFF to a trap containing protein hydrolysate or ammonium salt (Levi-Zada 2023). However, this baited trap usually captures relatively few of each sex, while ME attracts many more males (while not attracting females) (Kausar et al. 2022; our Fig. S1). Thus, there is an ongoing difficulty in exterminating PFF by mass trapping with the existing attractive baits. Due to this, the goal of management treatments today is to prevent the arrival of infected fruit at packing houses and minimize damage to fruit and vegetable.

Recently, there has been growing interest among researchers in developing a more effective and environmentally friendly pest control method known as push-pull (Pyke et al. 1987; Miller and Cowles 1990; Cook et al. 2007; Byers et al. 2018; Byers and Levi-Zada 2022). This method affects the insect with two forces: (1) repulsion from the host plant in the orchard, and (2) attraction by attractive baits in traps. The idea is to place traps with an attractant around the perimeter of the orchard, or at a suitable distance from the host plants, while applying repellents inside the orchard, particularly near the host plants. A fly outside the orchard will first encounter an attractive trap and might be caught. However, if the fly is not caught in the trap and moves into the orchard, it will be repulsed by the distributed repellents and then have more chances to be caught in the attractive traps. Another option is to apply a repellent in an infested area near the host plants of the plantation. At the same time, attractive traps are placed within the area but far enough from host plants to be relatively unaffected by the repellents. In the case of PFF, ME or protein hydrolysate, can be used as an attractant, to capture the pest before it enters the plot, or in traps within the plot but not too close to the host plants. We demonstrated, using computer models, that the push-pull method is significantly more effective than the mass trapping method alone, as it exerts two forces on the insect: repulsion and attraction, rather than attraction only (Byers et al. 2018; Byers and Levi-Zada 2022).

Therefore, to use the push-pull method on PFF, an attractant and a repellent, the latter of which has not yet been identified, are required. In recent years, there have been reports of attempts to find substances that would repel PFF, aiming to develop the push-pull method. However, the attempts to find a repellent were quite arbitrary and inadequate, with only a few fragrant essential oils or volatiles screened that might mimic or correspond to odors from non-host plants that the fruit fly has evolved to avoid in its natural habitat (Fouda et al. 2017). In fact, most of the volatiles were only tested under laboratory conditions (Tajdar et al. 2019). In the laboratory arenas or Y-tube olfactometers, the insects are presented at very close range to the source of putative bioactive volatiles, such that some are apparently attractive or repulsive. However, when these volatiles were released in the field over a much longer range (several meters), they had no observable behavioral activity (and no results were published). This is likely due to the comparatively high concentrations in the laboratory bioassay, which elicited statistically significant differences, but became diluted under field conditions to an inactive concentration. Therefore, we believe that field experiments are more appropriate and rigorous than laboratory tests, and thus likely to indicate biological activity that will lead to practical applications in IPM.

Compared to insect pheromones, where the source is known, identifying natural repellents of insects can be more challenging, as any such chemicals are likely derived from non-host plants, which are largely unknown to the researcher (Deletre et al. 2016). Therefore, identifying repellents for specific pest insects is a doubtful endeavor that often has relied on testing relatively few volatiles known as repellents of other species, with the chance that some might be repellent to the target pest (Guarino et al. 2013; Faleiro et al. 2022). A more comprehensive approach is the testing of many EOs of odiferous plants, as exemplified by the screening of 80 EOs with many volatile constituents to isolate repellents for the red palm weevil that counteracted their attraction to male-released aggregation pheromone (Levi-Zada et al. 2024).

Essential oils (EOs) are found in different plants and are extracted from the different parts of the plant: seeds, leaves, buds, stem bark, roots, and flowers (Yingngam 2022). Essential oil can be extracted by cold pressing or steam distillation from many plants, and each such oil will have a different chemical composition of dozens of substances (Berger 2010). Some EOs are relatively stable chemically and can be purchased from many commercial suppliers (Turek and Stintzing 2013). One of the functions of EOs in nature is to protect the plant against insects (Isman 2000). Essential oils have been widely used in plant protection, primarily as pesticides (Isman 2006; Adorjan and Buchbauer 2010; Ayllón-Gutiérrez et al. 2024). However, most studies published on EOs as repellents have focused on one volatile compound or on only a few EOs, while many constituents have not been tested, likely due to the difficulty of testing numerous combinations in field conditions. Our working assumption is that if we systematically scan many EOs, it is likely that there will also be volatile plant compounds among them that will repel the pest. Our objective here is to employ this methodology to investigate 82 EOs and their constituents that may counteract the attraction of male PFF to ME, as well as the attraction of both sexes to protein hydrolysate. Finding substances that repel PFF, and other fruit fly species attracted to ME, will be highly rewarding if this knowledge can be used to develop a push-pull control method. Our approach can also detect synergism among EOs and constituents in repellent activity, although synergism greatly complicates and can overwhelm with the tests required to isolate the important synergists.

## Materials and Methods

### Chemicals

Attractive PFF male lures comprised of 100 mg ME (> 98%, FCC, Sigma, Israel) impregnated on a disk (0.8 mm thick × 3 mm width) of dental cotton roll (size 3, Naot Medical, Israel). The disks were placed and heat-sealed in a PE (polyethylene) flat sleeve (0.11 mm thickness, Polysac, Israel) of 2.5 × 5 cm^2^ and were used as a positive control.

The same dispenser was used with two cotton disks in combination with various numbers of the 82 EOs: #1–77 Shifon cosmetics and raw materials, Ganei Yohanan, Israel; # 78 Eden Garden, San Clemente, CA, USA; #79–80 Orlife Global, Istanbul, Turkey; # 81–82 A. Fakhry & Co., Cairo, Egypt. The EOs used in field trials were randomly assigned to the following numbers: (1) Angelica seed, (2) Anise star, (3) Basil, (4) Bergamot, (5) Black pepper, (6) Cajeput, (7) Camphor white, (8) Cardamon, (9) Carrot seed, (10) Cedarwood Atlas, 11. Cedarwood Virginia, 12. Chamomile German, 13. Chamomile Roman, 14. Cinnamon bark, 15. Cinnamon leaf, 16. Cistus, 17. Citronella, 18. Clary sage, 19. Clove bud, 20. Coriander seed, 21. Cypress, 22. Desert Wormwood, 23. Elemi, 24. *Eucalyptus citriodora*, 25. *Eucalyptus radiata*, 26. Fennel, 27. Frankincense, 28. Galbanum, 29. Geranium, 30. Grapefruit white, 31. Helichrysum organic, 32. *Helichrysum*, 33. Ho-Wood, 34. Juniperberry, 35. Laurel leaf, 36. Lavender, 37. Lemongrass, 38. Lime, 39. *Litsea cubeba*, 40. Mandarin Red, 41. Manuka, 42. Marjoram, 43. Mastic, 44. Melissa, 45. *Mentha arvensis*, 46. Myrrh, 47. Myrtle, 48. Neroli, 49. Niaouli, 50. Nutmeg, 51. Oregano, 52. Orange (sweet), 53. Palmarosa, 54. Parsley seed, 55. Patchouli, 56. Peppermint, 57. Petitgrain, 58. Pine scotch, 59. *Ravensara aromatica*, 60. Ravintsara, 61. Rosemary, 62. Rosewood, 63. Sage, 64. Sandalwood West India, 65. Sandalwood Santalum spicatum, 66. Sibirian fir, 67. Silver fir, 68. Spearmint, 69. Tangerine, 70. Tea tree linalool, 71. Thyme linalool, 72. Thyme red, 73. Vetiver, 74. Wintergreen, 75. Yarrow, 76. Ylang-ylang complete, 77. Neem, 78. Catnip, 79. *Juniperus communis*, 80. *Curcuma longa*, 81. Fenouil, 82. Auroni male.

Other chemicals for syntheses, identification, and field testing of components of the two most repellent EOs found in the initial experiments were purchased: *β*-caryophyllene (≥ 80%, SAFC), *β*-linalool (97%, Fluka), benzyl benzoate (99%, TCI), sabinene (75%, Angene), *β*-pinene (99%, Alfa Aesar), eucalyptol (> 98%, Sigma), *α*-caryophyllene (96%, TCI), (+)-δ-cadinene (96%, TCI). Geranyl acetate (92%) was prepared by acetylation of geraniol in pyridine/acetic anhydride (Zada and Harel 2004). All solvents were purchased from Biosolve, BioLab, Israel. Silica gel 60 (70–230 mesh) was purchased from Mercury, Rosh HaAyin, Israel. Germacrene D (70% chemical purity), (*E*,*E*)-*α*-farnesene (60%), artemisia ketone (3,3,6-trimethyl-1,5-heptadien-4-one, 90%) and chamazulene (95%) were separated from Yarrow and Ylang-ylang EOs by liquid chromatography on silica gel 60 (70–230 mesh, Mercury, Rosh HaAyin, Israel) with n-hexane (Biosolve) and increasing amounts of diethyl ether (Biosolve). These components were re-chromatographed on a silica gel (200–425 mesh) column eluting with 1–3% ether in n-hexane to increase final purity.

### Chemical Analysis

The chemical constituents of the most repellent EOs, Yarrow and Yang-ylang, were characterized on an Agilent 7890 A GC interfaced with a split to an Agilent 5975 C MS detector and FID (Agilent Technologies, Santa Clara, CA, USA). Chemical separations were done on a nonpolar Rxi-5 Sil MS (Restek, Bellefonte, PA 16823, USA) column (30 m × 0.25 mm ID × 0.25 μm film) initially kept at 50° C for 5 min, then programmed at 10 °C/min to 230° C and held for 10 min. Analyses on chiral columns were performed using: (1) Rt-ɣDEXsa (Restek, Bellefonte, PA 16823, USA) column (30 m × 0.25 mm ID × 0.25 μm film) kept at 60° C for 1 min, then programmed at 2 °C/min to 200° C, and (2) Rt-β-DEXsm (Restek) column (30 m × 0.25 mm ID × 0.25 μm film) kept at 40° C for 0 min, then programmed at 5 °C/min to 220° C and held for 10 min. Additional analyses were done with a polar VF-23 (Varian Inc., Lake Forest, CA) column (30 m × 0.25 mm ID × 0.25 μm film) kept at 50° C for 5 min, then programmed at 10 °C/min to 230° C and held for 10 min. The column flow was split to both FID and MS detectors equally by an Agilent purged two-way effluent splitter, enabling qualitative and quantitative analyses simultaneously. Analyses on all columns were performed in the splitless mode with the split valve opened 1 min after injection and the MS m/z sweep range was 50–350 a.m.u. Column helium flow was 1.5 mL/min and the GC-MS inlet temperature was 230° C. Identification was done by using Wiley 8/NIST 2008 MS libraries, comparing retention times with literature data of retention indexes (RI), and authentic standards.

### Field Experiments

#### Locations

Tests were conducted in an orchard with different citrus fruit varieties (31.987035, 34.825336) under conventional pest control, and in a mango orchard with various mango varieties without routine pest control (31.982936, 34.828096). Both plots were infested with PFF.

#### Lures and Traps

In general, lures were prepared by dissolving 200 mg of each of the tested EOs with 300 mg of heavy paraffin oil (Romical, Israel). This solution was impregnated on two 3 mm disks of dental cotton roll, unless stated otherwise, and put inside sealed 2.5 × 5 cm^2^ PE sleeves (0.11 mm thick, PolySac Ltd., Kannot, Israel). Female and male baits contained 50 mL of hydrolyzed protein solution (Ceratrap, Bioiberica, Gadot-Agro, Israel) poured into a plastic open cup (4 cm diameter) and placed in the trap. Male baits contained 100 mg ME impregnated in one cotton roll and sealed in PE sleeve as described above. The evaporation of ME from this lure was tested in a wind tunnel at 25 °C (according to Levi-Zada et al. 2024) and the release rate was ~ 1.78 mg/d during 20 days. Traps included one type of the mentioned attractants and one 1 × 1 cm^2^ rubber dispenser with a killing agent, 2,2-dichlorovinyl dimethyl phosphate (DDVP, Hercon, distributed by OrganiSheli, Israel). The traps in all experiments were Decis (Bayer, Gadot-agro, Israel), a trap type designed for fruit flies, and recycled by washing in a dishwasher between experiments. Traps with each of the treatments (possible repellent with attractant) and a control treatment (only attractant) were hung randomly in each orchard row at a spacing of 10 m between nearby traps, and this arrangement was replicated at random in each of several adjacent rows separated by 10 m between rows, forming a grid of trap treatments. PFF were collected from traps at the end of reported test periods and counts of each sex were recorded in the laboratory.

#### Trap Shutdown

When a repellant EO is released in the trap along with an attractant (ME), then the attraction (i.e., capture rate) of this trap for PFF, or any other *Bactrocera* species that is attracted to ME, will be significantly lower than the catches of control traps with ME alone. The rate of decrease in captures in a trap with attractant and repellent compared to the attractive control trap is measured by a parameter called *trap shutdown* (ranging from 0%, no shutdown effect, to 100% for total repulsion) and is calculated as follows (Faleiro et al. 2022; Levi-Zada et al. 2024):

$$\%\;\mathrm{trap}\;\mathrm{shutdown}=100-\mathrm{TR}^\ast100/\mathrm{TA}$$

where TR = flies captured in traps with repellents + Attractant; TA = flies captured in traps with attractant only.

#### Testing EO’s #1–82

The 82 EOs were tested in two successive field experiments of 7 days each in October-November 2023, the first experiment in the mango orchard on 26.10–1.11.2023 (I) and the second (II) in the citrus orchard on 6-13.11.2023. The EOs were divided into five major groups of 16 or 17 EOs each in the numbered sequence (each group was a different treatment). Dispensers were prepared as described above. EOs of the same group were placed together in a 5 × 5 × 5 cm^3^ metal screen cage and placed inside a trap along with a dispenser of ME and killing agent DDVP (*N* = 5 replicates for each treatment group). Control traps included ME lure and DDVP dispenser only. Details regarding this trial are given in Table S1.

#### Testing EO’s 66–82

Seventeen EOs of group #66–82, that was the most repellent of the five major groups tested above, were divided into four sub-groups of four to five EOs each and tested in the mango orchard on 15-22.11.2023. One treatment contained all the EOs #66–82 together, and the other treatments contained each of the sub-groups alone (EOs # 66–69, 70–73, 74–77, and 78–82). Each treatment was displayed in traps along with ME bait and DDVP dispenser. Control traps included ME lure and DDVP dispenser only (*N* = 5). In the same way, the groups of EOs 1–16 and EOs 17–32, with significant but less trap shutdown, were tested in the citrus orchard on 28.11–5.12.2023 and 12-19.12.2023, respectively. EOs # 33–48 group was tested similarly in the mango orchard on 7-14.3.2024. The results showed that the subgroups had only moderate to weak repellency, and thus were not tested further (see Figs. S2-S4). Details of these trials are given in Table S1.

#### Testing EOs Sub-Group #74–77

Testing the five subgroups of EOs #66–82 showed that the EOs #74–77 group was the most repellent. Thus, its four EOs were tested together compared to each EO alone in the mango plot on 28.11–5.12.2023. One treatment included four dispensers of 200 mg of each EO, and the other treatments included each EO alone (four dispensers of 200 mg of EO in each trap). All treatment and control traps included ME lure and DDVP dispenser (*N* = 5). Details of this trial are given in Table S1.

#### Testing EOs #75 (Yarrow) and #76 (Ylang-ylang)

Since EOs #75 and #76 were the most repellent above, an experiment was conducted in the mango orchard (12-19.12.2023) that had five treatments: (1) four dispensers of 200 mg each of EO #75; (2) four dispensers of 200 mg each of EO #76; (3) two dispensers of 200 mg each of #75 plus two dispensers of 200 mg each of #76; (4) eight dispensers of 200 mg each of #76; and (5) control. Each trap, including control, contained ME and DDVP dispensers. A similar experiment was conducted in the citrus orchard (26.12.2023-2.1.2024), but did not include the double-dosage treatment of #76 (*N* = 6 replicates per treatment). Details of these trials are given in Table S1.

#### Repellent EO Dosage Effect

Different dosages of EO #76 (Ylang-ylang) were dissolved (1:1.5 W/W) in heavy paraffin oil. Value 1X (the following treatment 4) is the EO volatile release of four PE dispensers (0.11 mm thick) of 5 × 2.5 cm^2^ surface area, that were used in the previous and subsequent experiments. All other X values are related to this 1X dispenser. The EO amount does not affect the release rate, but only the lifespan of the dispenser (Cha et al. 2017). Thus, five treatments were prepared as follows: (1) 25 mg EO in one PE sleeve of 1.25 × 1.25 cm^2^ (0.03125X); (2) 50 mg EO in one PE sleeve of 1.25 × 2.5 cm^2^ (0.0625X); (3) 200 mg EO in one PE sleeve of 5 × 2.5 cm^2^ (0.25X); (4) four PE sleeves of 5 × 2.5 cm^2^ with 200 mg EO each (1X); and (5) sixteen 5 × 2.5 cm^2^ PE sleeves of 200 mg EO each (16X). Each trap contained ME and DDVP dispensers and was displayed randomly as above in the mango orchard (28.12.23–4.1.2024; *N* = 6). Details of this trial are given in Table S1.

#### Testing the Effect of EOs # 75 + 76 on the Shutdown of Female Catches

Three experiments were conducted in the mango orchard, each containing the following treatments. Experiment I (14-28.3.2024): (1) 50 mL food bait (Ceratrap) as mentioned above; (2) food bait and two dispensers of 200 mg each of EOs 75 and 76, and (3) DDVP dispenser alone (*N* = 6). Experiment II (12-19.12.2024): (1) 50 mL food bait as above; (2) food bait with two dispensers of 200 mg each of EOs 75 and 76; (3) food bait with four dispensers of 200 mg each of EO 75; (4) food bait with four dispensers of 200 mg each of EO 76; and (5) DDVP dispenser alone (*N* = 5). All traps contained DDVP dispensers as a killing agent. Experiment III (19.12.2024-8.1.2025): same as Exp. II but no DDVP tested alone (*N* = 5). Details of these trials are given in Table S1.

#### Testing EO #75 - Yarrow - and its Major Components

Two field experiments were conducted in the mango orchard on 28.10.2024-4.11.2024 and 14-21.11.2024. The composition and ratio of each of the major components (> 4%) of EO #75 were determined by GCMS-FID analysis (Table 1). The treatments of the experiments on 28.10.2024-4.11.2024 consisted of (1) Four dispensers with 200 mg of EO #75; (2) Four dispensers that contained 560 mg of a mixture of sabinene, β-pinene, eucalyptol, and *β*-caryophyllene (200, 160, 40, 160 mg respectively), four dispensers of artemisia ketone (40 mg), germacrene D (120 mg) and chamazulene (80 mg), each separately; (3) 800 mg sabinene in four dispensers; (4) 800 mg *β*-pinene in four dispensers; (5) 800 mg eucalyptol in four dispensers; (6) 800 mg *β*-caryophyllene in four dispensers; (7) control (100 mg ME); and (8) DDVP alone. The EO and the four major components of the mixture in treatment 2 above, and artemisia ketone, were dissolved in paraffin oil 1:1.5 w/w, impregnated in a cotton roll disk and placed inside PE sealed sleeves. Germacrene D and chamazulene were placed neat on a cotton roll in the PE sleeve as stated above. Each trap, including control traps, contained ME and DDVP dispensers (*N* = 6). Details of this trial in Table S1.

The treatments of the experiment that was conducted on 14-21.11.2024 consisted of the same treatments as above except that treatments 3–6 contained (3) 400 mg artemisia ketone; (4) 400 mg germacrene D; (5) 400 mg chamazulene; and (6) 400 mg *β*-caryophyllene, each divided among four dispensers. Although amounts of these compounds were reduced, release rates from the plastic dispensers remained unchanged.

#### Testing EO #76 – Ylang-ylang - Compared to its Major Components

Two field experiments were conducted in the mango orchard (8-15.10.2024 and 2-9.2.2025). The composition and mixture ratio of the major components (> 4%) of EO #76 (Ylang-ylang) were determined by GC-MS/FID analysis (Table 1). The experiments of 8-15.10.2024 included the following treatments: (1) 100 mg ME; (2) four dispensers of 200 mg of EO #76; (3) a total of 800 mg of a mixture of eight major components in ratios similar to that of the EO [Linalool (80 mg), geranyl acetate (80 mg), *β*-caryophyllene (200 mg) and benzyl benzoates (80 mg)] were dissolved in paraffin oil 1:1.5 w/w and were combined and impregnated in four dispensers; Germacrene D (120 mg) loaded neat on four dispensers, *α*-caryophyllene (80 mg), *δ*-cadinene (40 mg) and (*E*,*E*)-*α*-farnesene (120 mg) were mixed neat, and their mixture was divided on four dispensers. This was due to their low vapor pressures and a long purification process; (4–7) four dispensers of 200 mg (total 800 mg) of each pure major component of the EO - *β*-linalool, geranyl acetate, *β*-caryophyllene and benzyl benzoate - each impregnated in four cotton roll disks (in PE sleeves) with prior dissolving in paraffin oil 1:1.5 w/w. Each trap, including control traps, contained ME and DDVP dispensers (*N* = 6).

The experiment, conducted on 2-9.2.2025, consisted of treatments 1–2 as above. Treatments 3–5 contained four dispensers of 50 mg (total 200 mg) of *α*-caryophyllene, (*E*,*E*)-*α*-farnesene, and (+)-*δ*-cadinene, each as a different treatment. Each trap, including control traps, contained ME and DDVP dispensers (*N* = 5).

#### Testing the Effect of Artemisia Ketone on the Shutdown of Female Catches

This experiment was conducted in mango orchard for three weeks 16.1.2025-6.2.2025 and contained the following treatments: (1) 50 mL food bait (Ceratrap) as mentioned above; (2) 50 mL of food bait plus four dispensers of 200 mg of artemisia ketone impregnated on a cotton disk in a PE sealed sleeve; and (3) DDVP dispenser alone (*N* = 6).

### Statistics

Means (± SE) of captured flies (counts) on traps of treatments and controls were calculated in Excel (Microsoft, Redmond, WA). Statistical analysis was conducted by R software for Windows version 4.4.0 (R Core Team 2024). Statistical differences between counts on traps were analyzed by General Linear Models (GLM) with Poisson regression followed by Tukey-Sidak post-hoc tests (Mangiafico 2016).

## Results

**Fig. 1:**
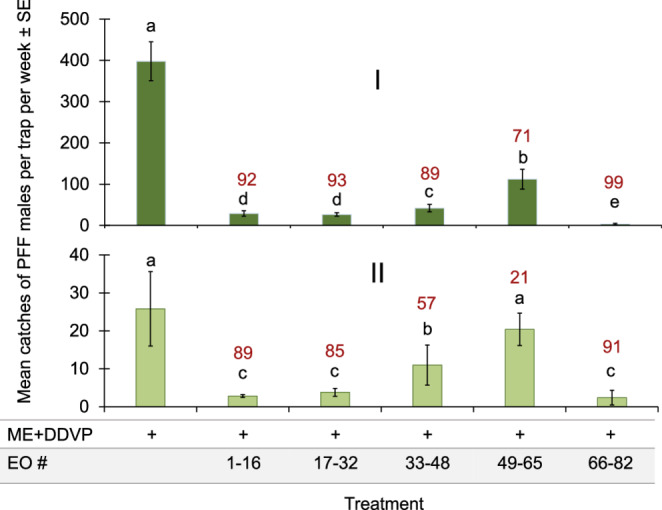
Mean catches of PFF males by ME baits combined with EO #1–82 divided into five groups per trap per week ± SE. I: in mango orchard (26.10–1.11.2023), II: in citrus orchard (6-13.11.2024). Bars of mean catch per trap for each of six treatments (*N* = 5 replicates/treatment) in each graph with letters in common are not significantly different (GLM-Poisson regression with Tukey-Sidak post-hoc tests for multiple comparisons, α = 0.05). The number above each column (in red) represents the trap shutdown (%) for ME + EO bait compared to the control

We tested all the EOs in our inventory in two identical field trials, one in a mango orchard and the second in a citrus orchard. The results of the two experiments show that EOs #66–82 caused a shutdown of 99% of ME traps in the mango plot and 91% in the citrus plot. This group was better than all other groups tested in the same experiments, though not always significantly different from the other groups (Fig. 1).

**Fig. 2:**
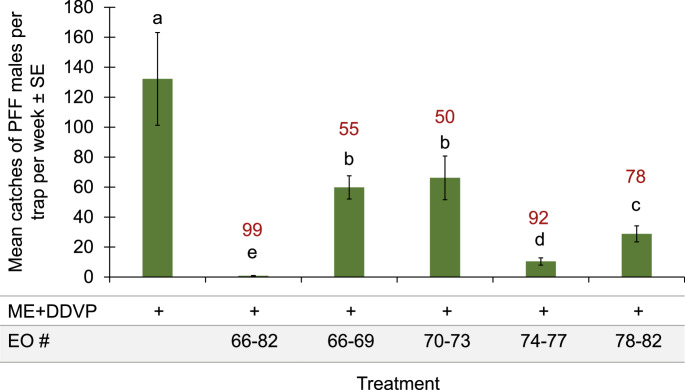
Mean catches of PFF males by ME baits combined with EOs #66–82 together and divided to 1–16, 17–32, 33–48 and 66–82 (15-22.11.2023) in mango orchard. Bars of mean catch per trap for each of the six treatments (*N* = 5 replicates/treatment) with letters in common are not significantly different (GLM with Tukey-Sidak post-hoc tests for multiple comparisons, α = 0.05). The number above each column (in red) represents the trap shutdown (%) for ME + EO bait compared to the control

In the following experiments we tested the other EOs groups 1–16, 17–32, and 33–48 (Figs. S2-S4), but they were not as strongly repellent as group 66–82, which reduced trap catches when divided into four sub-groups (Fig. 2). Therefore, we continued focusing on group 66–82 only. The group of EOs # 74–77 gave the highest trap shutdown of 92% when the EOs were combined with ME in the traps, and it was statistically similar in catches with all the EOs of 66–82 (Fig. 2). DDVP was merely a killing agent used in nearly all experiments in all traps.

**Fig. 3:**
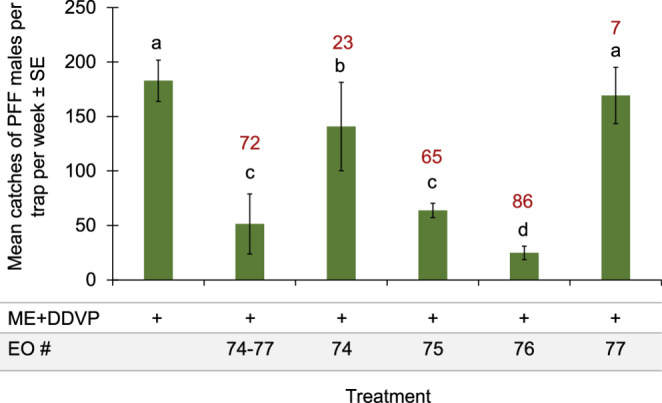
Mean catches of PFF males in the mango orchard by ME baits combined with EOs #74–77 together and each EO alone (28.11–5.12.2023). Bars of mean catch per trap for each of six treatments (*N* = 5 replicates/treatment) with letters in common are not significantly different (GLM with Tukey-Sidak post-hoc tests for multiple comparisons, α = 0.05). The number above each column (in red) represents the trap shutdown (%) for ME + EO bait compared to the control

In the next experiment conducted in the mango orchard (28.11–5.12.2023), we divided the sub-group of EOs # 74–77 into four EOs, and two of its EOs, which were tested alone, # 75 and # 76, gave the same level of low catches in combination with ME bait as did the sub-group 74–77 (Fig. 3).

**Fig. 4:**
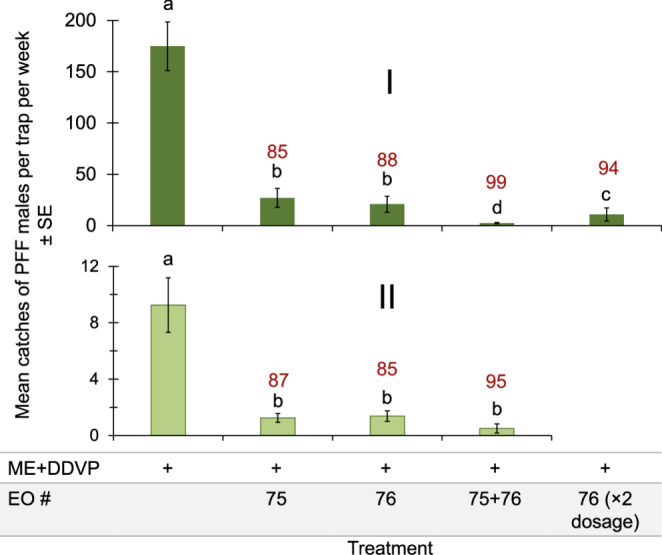
Mean catches of PFF males by ME baits combined with EO #75 (Yarrow), EO #76 (Ylang-ylang) and both EOs in mango (I, 12-19.12.2023, *N* = 6) and citrus (II, 26.12.2023-2.1.2024, *N* = 8) orchards. Bars of mean catch per trap for each graph with letters in common are not significantly different (GLM with Tukey-Sidak post-hoc tests for multiple comparisons, α = 0.05). The number above each bar (in red) represents the trap shutdown (%) for ME + EO bait

We continued by focusing on the two repellent EOs # 75 and #76. These EOs are Yarrow and Ylang-ylang, and they were tested together and individually, in combination with ME bait, in both mango and citrus orchards (Fig. 4). We observed very low catches in all treatments, especially when both EOs were combined. The double dosage of EO #76 reduced the catches similar to those of the two EOs together (Fig. 4I).

**Fig. 5:**
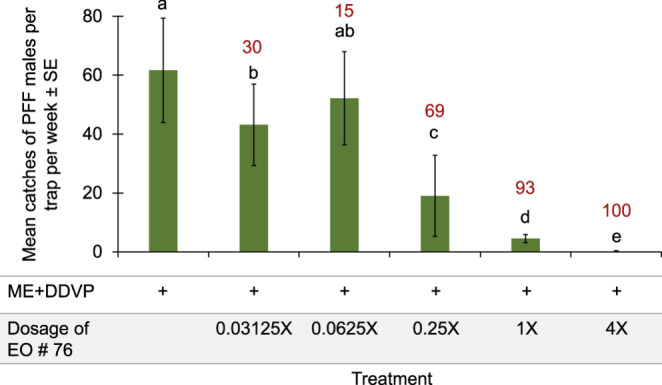
Mean catches of PFF males in mango orchard by ME baits combined with EO Ylang-ylang (#76) in five increasing release rates (0.03125X to 4X), while 1X is the release rate used in most experiments with this EO (28.12.2023-4.1.2024). Bars of mean catch per trap for each of six treatments (*N* = 6 replicates/treatment) with letters in common are not significantly different (GLM with Tukey-Sidak post-hoc tests for multiple comparisons, α = 0.05). The number above each column (in red) represents the trap shutdown (%) for ME + EO bait compared to the control

In the next experiment, we tested how the release rate of EO # 76 (Ylang-ylang) affects the trap shutdown of male catches on ME baits (28.12.2023-4.1.2024). The release rate of volatiles from PE plastic dispensers is determined by the thickness of the PE sleeve and its surface area (Cha et al. 2017). Increasing release rates of the repellent Ylang-ylang EO, dissolved in paraffin oil in appropriate dosage ratios, increased the trap shutdown of male catches progressively to 100% shutdown at sixteen dispensers of 200 mg of EO in 5 × 2.5 cm^2^ PE sleeves (4X Fig. 5), while four dispensers of 200 mg (1X Fig. 5), used in most experiments testing this EO, gave low catches with shutdown of about 93%.

**Fig. 6:**
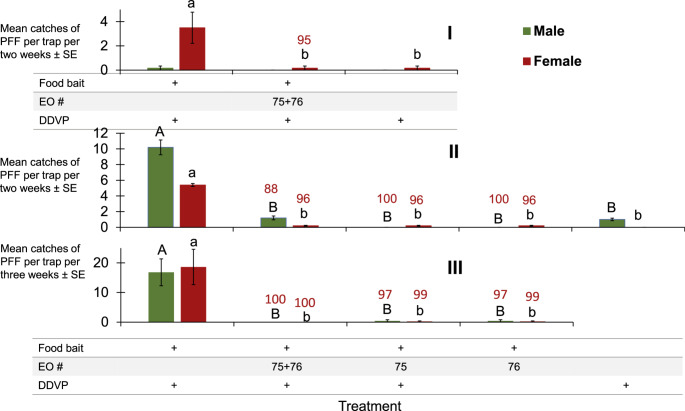
Mean catches of PFF males and females in mango orchard by food-baited traps I. with EOs 75 + 76 (14-28.3.2024; *N* = 6). II & III with EOs 75 + 76 and each alone (12-19.12.2024, and 19.12.2024-8.1.2025, respectively; *N* = 5). For each graph and sex, bars of mean catch per trap with letters in common are not significantly different (GLM with Tukey-Sidak post-hoc tests for multiple comparisons, α = 0.05). The number above each column (in red) represents trap shutdown (%) for food bait + EO relative to same-sex captures in the corresponding control

The next experiments were conducted to test whether the two EOs # 75 and #76, which provided excellent shutdown for traps with ME baits that attract only males, would also show a trap shutdown using a food bait that attracts both males and females. Females are the primary target for controlling pests, as they cause the most damage. Thus, we repeated these experiments three times. The results (Fig. 6) show that EO’s #75 and #76 cause trap shutdown in the 88 to 100% range for PFF males and females attracted to food odors.

The following experiments were designed to find which components of the repellent EOs were responsible for their repellency. The experiments were divided so that in each experiment the repellent EO and some of its pure components were tested. In all experiments, traps with ME alone served as a control.

**Fig. 7:**
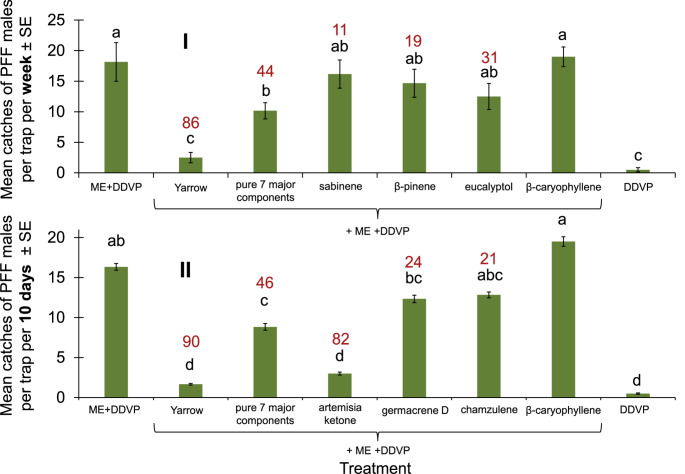
Mean catches of PFF males by ME baits with EO Yarrow (#75) pure components per trap. (I) in mango orchard 28.10.2024-4.11.2024, (II) in mango orchard 14-21.11.2024 with different components. Bars of mean catch per trap for each of eight treatments (*N* = 6 replicates/treatment) in each graph with letters in common are not significantly different within an experiment (GLM with Tukey-Sidak post-hoc tests for multiple comparisons, α = 0.05). The number above each column (in red) represents trap shutdown (%) for each treatment compared to the corresponding control (ME)

In the next two experiments, the mixture of the seven pure ingredients in appropriate ratios as that in the EO only gave 44–46% trap shutdown as that for the Yarrow EO (Fig. 7). Of the seven major ingredients in Yarrow oil (#75), only the artemisia ketone gave a high degree of 82% trap shutdown, not different from that of Yarrow EO itself (Experiment II, Fig. 7).

**Fig. 8:**
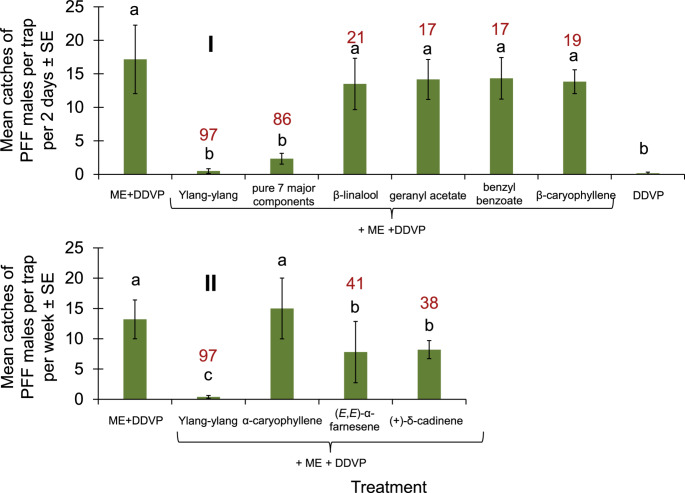
Mean catches of PFF males by ME baits with EO Ylang-ylang (#76) pure components per trap. (I) in mango orchard 8-15.10.24, (II) in mango orchard 2-9.2.2025 with different components. Bars of mean catch per trap for each of the treatments (*N* = 6 replicates/treatment) with letters in common are not significantly different within an experiment (GLM with Tukey-Sidak post-hoc tests for multiple comparisons, α = 0.05). The number above each column (in red) represents trap shutdown (%) for each treatment compared with the corresponding control (ME)

On the other hand, none of the seven main substances identified in Ylang-ylang EO #76 gave the same trap shutdown as that of the EO itself (Fig. 8). However, in this essential oil, the mixture of the seven pure ingredients tested in the same ratios as they make up the oil caused trap shutdown almost as much as the EO itself, 86% compared to 97% in the EO, which were not statistically different (Fig. 8).

**Fig. 9:**
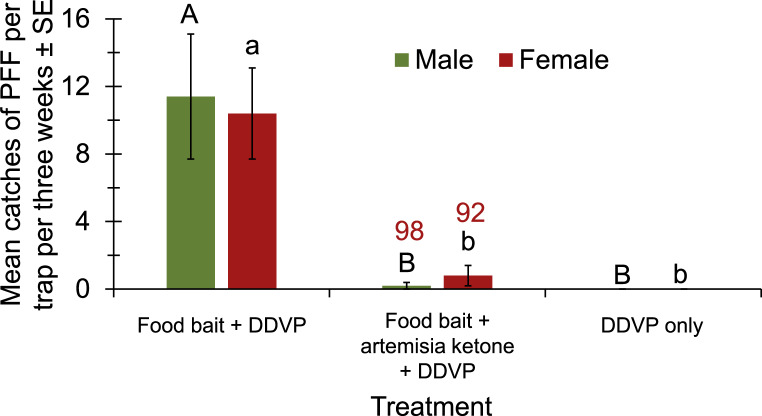
Mean catches of PFF males and females per trap with either food bait, food bait plus artemisia ketone, or a blank trap, in a mango orchard (16.1.2025-6.2.2025). Bars of mean catch per trap for treatments (*N* = 5 replicates/treatment) with letters in common within each sex are not significantly different (GLM with Tukey-Sidak post-hoc tests for multiple comparisons, α = 0.05). The number above bars (in red) indicates the degree of trap shutdown relative to capture of the same sex in traps with control (food bait)

The last experiment in the field was designed to test the degree of shutdown of food bait traps by artemisia ketone, the repellent ingredient we found in Yarrow oil. The results indicate that this component of Yarrow EO causes the shutdown of food-baited traps, resulting in a 98% reduction in the number of males and 92% reduction in the number of female captured (Fig. 9).

The major constituents with relative quantities above 4% of the two essential oils that caused significant shutdown in traps with attractants of PFF males and females were analyzed with GCMS-FID using MS libraries, injections of authentic standards to four types of GC columns (a-polar, polar, and two chiral), and comparison with RI’s information of the literature (Acree and Arn 2004; Babushok et al. 2011; Adams 2017). These analyses show that Yarrow (EO # 75) and Ylang-ylang (EO # 76) EOs share two major components (> 10%), β-caryophyllene and germacrene D, and two minor components, α-caryophyllene and δ-cadinene (Table 1). The other identified components, which are known in the literature, are minor, and not found in both EOs; therefore, they were not combined in the field tests. Some components are volatile, while others are semi-volatiles, so their release rates may differ if they are impregnated in the same slow-release device (dispenser). Thus, when we tested constituents alone, we mixed the more volatile ones in the dispenser with paraffin oil to achieve a release rate that was approximately the same for all components (Levi-Zada et al. 2024).

**Table 1:** Identified major components (> 4%) and their relative percentages in EOs of repellent Yarrow and Ylang-ylang determined by GC-MS/FID analysis on different capillary GC columns

EO major component identified by GCMS (> 4%)	Relative abundance percentage in Yarrow EO #75 (%)^1^	Relative abundance percentage in Ylang-ylang EO #76 (%)^1^	Retention index (RI) on GC column			
			Rxi-5Sil MS^2^ (a-polar)	VF23 (polar)	Rt-γDexsa (chiral 1)	Rt-βDexsm (chiral 2)
sabinene	17		974	1132	1038/1041^3^	1014/1026^3^
β-pinene	15		978	1109	1051	1031
eucalyptol	4		1033	1261	1120	1079
artemesia Ketone	5		1059	1408	1187	1103
(±)-β-linalool		6	1100	1569	1276	1223
geranyl acetate		7	1379	1671	1520	1454
β-caryophyllene	12	16	1429	1825	1538	1451
α-caryophyllene	2^4^	5	1465	1760	1547	1487
germacrene D	11	11	1490	1796	1566	1511
(*E*,*E*)-α-farnesene		11	1507	1770	1579	1538
δ-cadinene	1^4^	5	1525	1776	1579	1571
chamazulene	7		1747	N.A	1893	1868
benzyl benzoate		7	1785	N.A.	2007	1933

^1^The ratios are relative to all components including minor ones found in each EO as determined by FID detector

^2^Compared with available authentic standards, NIST database and literature ( Acree and Arn 2004; Babushok et al. 2011; Adams 2017 )

^3^ Two enantiomers

^4^Not considered as a major component in this EO. Shown as additional information

## Discussion

Attractive semiochemicals of pest insects, specifically pheromones, have been used in agriculture for several decades to help reduce the frequency of pesticide applications, thereby mitigating pest resistance and environmental toxicity. However, some pheromones of pests do not perform as expected and are therefore not being used in IPM, despite the many efforts invested in isolating, identifying, synthesizing, and proving their biological activity.

The peach fruit fly (PFF) *B. zonata* is an important invasive species causing substantial losses to the horticulture industry worldwide. The male and female airborne volatiles have mainly been identified, but their release from traps in nature has not proven attractive to the pest (Levi-Zada et al. 2020). The volatile chemicals isolated and identified from species of *Bactrocera* are generally characteristic of the genus; for example, females usually contain spiroacetals and fatty acid esters, males have pyrazines, and both sexes have fatty acid acetamides (Fletcher and Kitching 1995; Symonds et al. 2009; Levi-Zada et al. 2020). Yet, it has proven difficult to show that the volatile compounds of *Bactrocera* fruit fly species are active in monitoring traps. Thus, relatively little is known about the functions and long-range attractiveness of the putative pheromones in most *Bactrocera* species. Except in the case of olive fruit fly, *B. olea*, sex pheromones are not used in the IPM practice of *Bactrocera* species (Heath et al. 1999). However, fortunately, males of *Bactrocera* fruit flies respond strongly to synthetic lures of ME, cue-lure (4-(p-acetoxyphenyl)−2-butanone) and raspberry ketone (4-(4-hydroxyphenyl)butan-2-one) (Howlett 1912, 1915); Chambers 1977; Qureshi et al. 1976; Tan et al. 2014). ME is found in over 450 plant species in nature and acts as a precursor to male fruit fly sex pheromonal components in the rectal gland of a few *Bactrocera* species (Tan and Nishida 2012). In *B. zonata*, ME is used for monitoring (Tan et al. 2014), the male-annihilation technique (MAT), and the sterile insect technique (SIT) (Gazit et al. 2024). Still, although ME manipulates the behavior of the males, the females are not attracted but are the ones who cause the damage to the fruits. Another bait used widely for *B. zonata* and other *Bactrocera* fruit flies is protein food baits, which attract both sexes, but the attraction to these food baits is much weaker than to ME (Fig. S1).

The “push-pull” pest control method (Khan et al. 2011; Byers and Levi-Zada 2022) was reported for the notorious citrus and mango pest, the Mexican fruit fly *Anastrepha ludens*, and other fruit flies (Birke et al. 2020). The method was tested with anastrephamide, a deterrent that serves as the host marking pheromone, and the “Pull” agent was the bait GF120™. This protein bait acts as a general attractant for fruit fly pests, combined with an insecticide product containing the active ingredient Spinosad. However, Birke et al. (2020) suggested that their push-pull system requires a more effective attractant to succeed. Unfortunately, individuals of species of *Anastrepha* genus are not attracted to ME (Tan et al. 2014).

We believe the push-pull technique is especially needed in cases where the pest pheromone or attractants are relatively weak and do not achieve a satisfactory level of control. However, the problem with push-pull development is that we need a good attractant and a repellent. The attractant could be a pheromone that is isolated from the insect or a plant material such as ME, but repellents are usually not from the insect or host plant but likely from non-host and exotic odiferous plants that in effect mimic the odor of the toxic/unsuitable non-host (Levi-Zada et al. 2024). Since plants have secondary compounds in their EOs, it is possible that some plants contain or produce repellents to protect themselves from herbivorous pests, such as *B. zonata*. Our hypothesis is that nearly every herbivorous insect is repelled by some plants and their EOs, so a repellent could be one compound or several compounds with additive or synergistic properties.

Indeed, our work shows that by systematically screening a relatively large number of commercial EOs, the likelihood increases that repellents will be isolated that can cause the shutdown of traps (Fig. 1). Then, these repellents can be applied in formulations to protect host plants from insect herbivore infestations. In the present case, we were able to isolate two EOs, Yarrow EO and Ylang-ylang EO, which dramatically reduced catches in traps containing male baits of ME (Figs. 2, 3 and 4) and male and female food baits (Fig. 6). We were then able to show that Yarrow EO has one component, artemisia ketone, that provides a high-level of trap shutdown of ME for males (Fig. 7). In the case of Ylang-ylang EO, the mixture of more than one component is needed to cause significant trap shutdown of males, indicating a repellent synergism among components (Fig. 8) (Nerio et al. 2010).

The artemisia ketone component caused the shutdown of traps with food baits that attracts both males and females (Fig. 9). Artemisia ketone is the major constituent of the EOs of many *Artemisia* plants. Radulović et al. (2013) showed that artemisia ketone exhibits a stronger free radical scavenging effect and a stronger antimicrobial activity than other monoterpenes in *Artemisia annua* L., which are known as traditional herbal medicine for malaria and can fight parasites and bacteria. Artemisia ketone was also reported as a contact toxicant to adults of the German cockroach, *Blattella germanica* L. (Blattodea: Ectobiidae) that is probably correlated to its capability to inhibit acetylcholine esterase (Yeom et al. 2015).

*Achillea millefolium* L., commonly known as Yarrow, is an insect repellent. Extracts of Yarrow have been tested for repellency in the laboratory against yellow fever mosquito *Aedes aegypti* (L.) and showed that the extract exhibited a similar repellency as the reference substances N, N-diethyl-m-toluamide and N, N-diethyl-mandelic acid amide (Thorsell et al. 1998). Yarrow EO also showed repellent activity towards blacklegged tick *Ixodes scapularis* (Say) nymphs (Pickett et al. 2023) and *Aegorhinus nodipennis* (Hope) (Coleoptera: Curculionidae) (Tampe et al. 2015).

*Cananga odorata* L. (Ylang-ylang) is known as a mosquito repellent for: (i) *Aedes aegypti* (L.), (ii) *Culex quinquefasciatus* (Say), and (iii) *Anopheles dirus* (Peyton & Harrison) (Siriporn and Mayura 2012; Soonwera 2015; Wathoni et al. 2018). Besides the repellent activity against mosquitoes, the EO of *C. odorata* leaves exhibited repellent activity against *Tribolium castaneum* (Herbst), the red flour beetle, a pest of stored products (Olivero-Verbel et al. 2013). This EO was shown in the study to have the strongest repellent effect against *T. castaneum* compared to four other tested essential oils and one “commercial repellent” (IR3535) of unknown structure and source.

Depending on their geographic source, an EO may have a variable composition, both qualitative and quantitative. The composition depends on the cultivar, climate, and the extraction method. Thus, finding one repellent compound, such as artemisia ketone, is an achievement. In Ylang-ylang EO, no single component was found to cause shutdown of traps, suggesting an interaction of compounds. Luckily, Ylang-ylang oil is a relatively inexpensive commercial oil compared to Yarrow oil and its single repellent component, artemisia ketone, so in the case of Ylang-ylang, it may be more economical to apply this EO with the same or similar composition. Since EOs contain volatile and semi-volatile ingredients, it is important to select a proper release device and/or formulation that releases the repellents at a consistently effective rate in the field for an extended period.

Using these EOs it will be possible to increase the efficiency of the work that has been done for many years by researchers on repellents. We believe that combining repellent essential oils within an IPM push-pull method, gaining momentum worldwide, will be an effective way to reduce damage from fruit flies.

## Electronic Supplementary Material

Below is the link to the electronic supplementary material.

ESM 1 (DOCX 159 KB)

## Data Availability

No datasets were generated or analysed during the current study.
