# Supplementary material for: Repelling Fruit Flies with Essential Oils and Their Components: the Peach Fruit Fly Bactrocera zonata
Source: J Chem Ecol. 2025 Aug 9;51(4):81. doi: 10.1007/s10886-025-01628-9 (PMC12335404; doi:10.1007/s10886-025-01628-9)
Supplement: Supplementary file 1 — (DOCX 159 KB) [file 10886_2025_1628_MOESM1_ESM.docx]

**Supplementary**

**Repelling Fruit flies with essential oils and their components: The Peach fruit fly *Bactrocera zonata***

Anat Levi-Zada*, Sara Steiner, Daniela Fefer, John A. Byers

Institute of Plant Protection, Department of Entomology-Chemistry unit, Agricultural Research Organization, Volcani Institute, Rishon LeZion, Israel.

Corresponding author: [anatzada@volcani.agri.gov.il](about:blank)


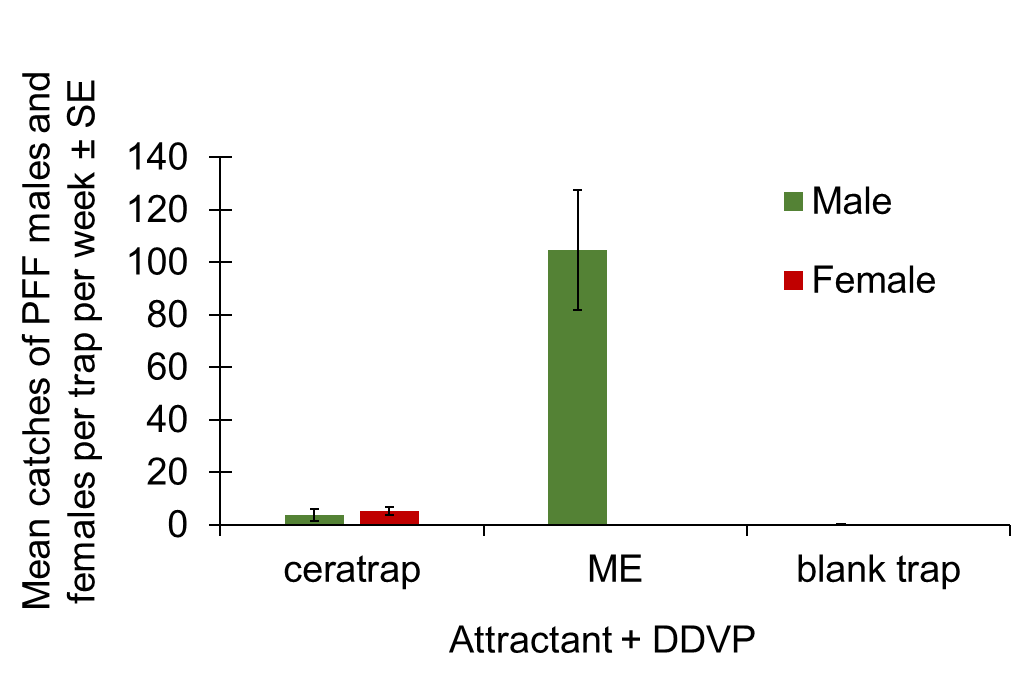


Fig. S1. Mean catches of Peach fruit fly (PFF) males and females attracted to traps (Decis) with 50 mL Ceratrap food bait compared to 100 mg methyl eugenol bait (ME) traps in mango orchard (16-23.1.2025) ± SE, N=5 replicates per treatment.


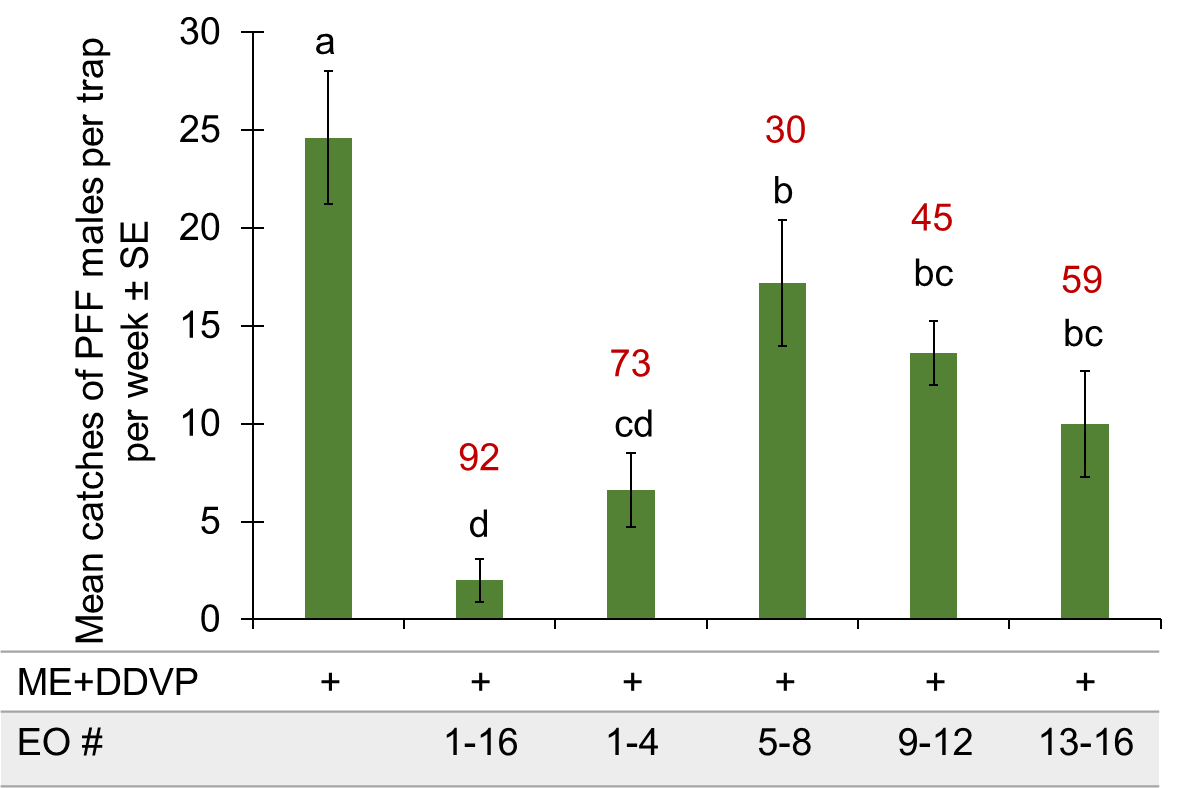


Fig. S2. Mean catches of PFF males by ME baits combined with EO #1-16 and sub-groups in citrus orchard (28.11-5.12.2023) per trap per week ± SE (N=5 replicates per treatment). Bars with letters in common are not significantly different (GLM-Poisson regression with Tukey-Sidak post-hoc tests for multiple comparisons, α = 0.05). The number above each column (in red) represents the trap shutdown (%) for this bait and EO.


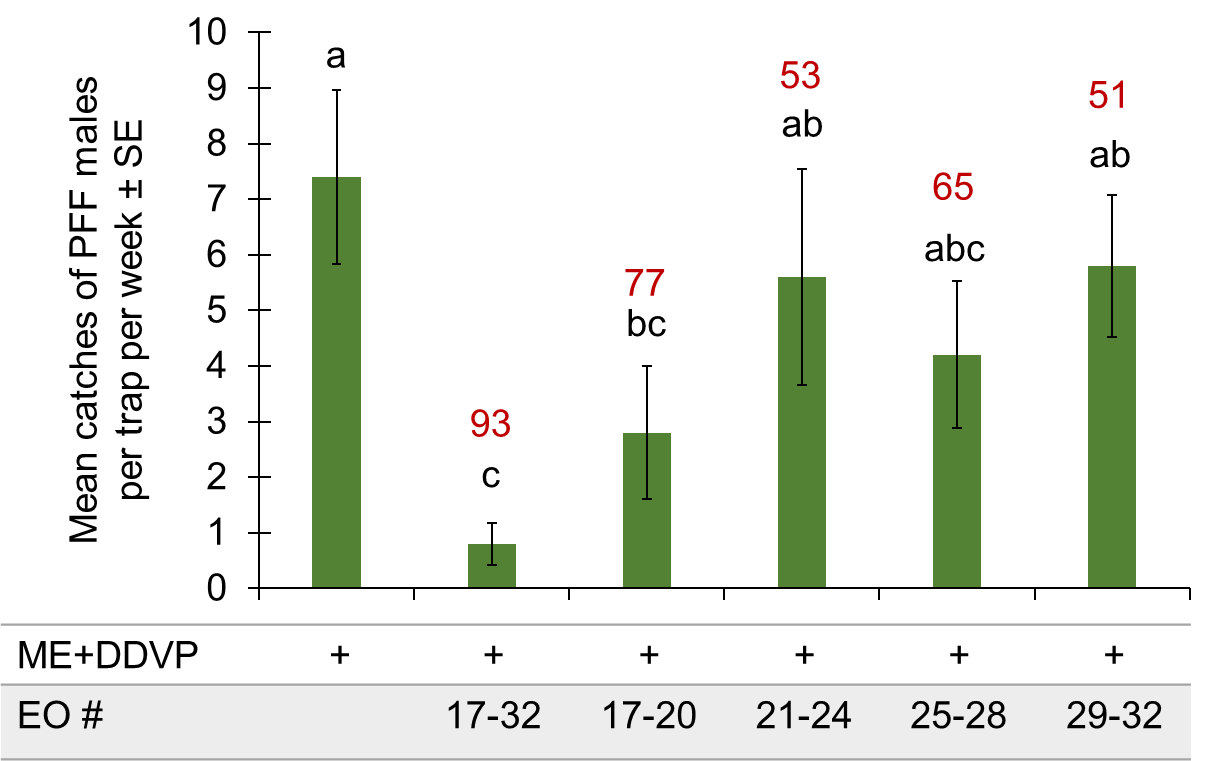


Fig. S3. Mean catches of PFF males by ME baits combined with EO #17-32 and sub-groups in citrus orchard (12-19.12.2023) per trap per week ± SE (N=5 replicates per treatment). Bars with letters in common are not significantly different (GLM-Poisson regression with Tukey-Sidak post-hoc tests for multiple comparisons, α = 0.05). The number above each column (in red) represents the trap shutdown (%) for this bait and EO.


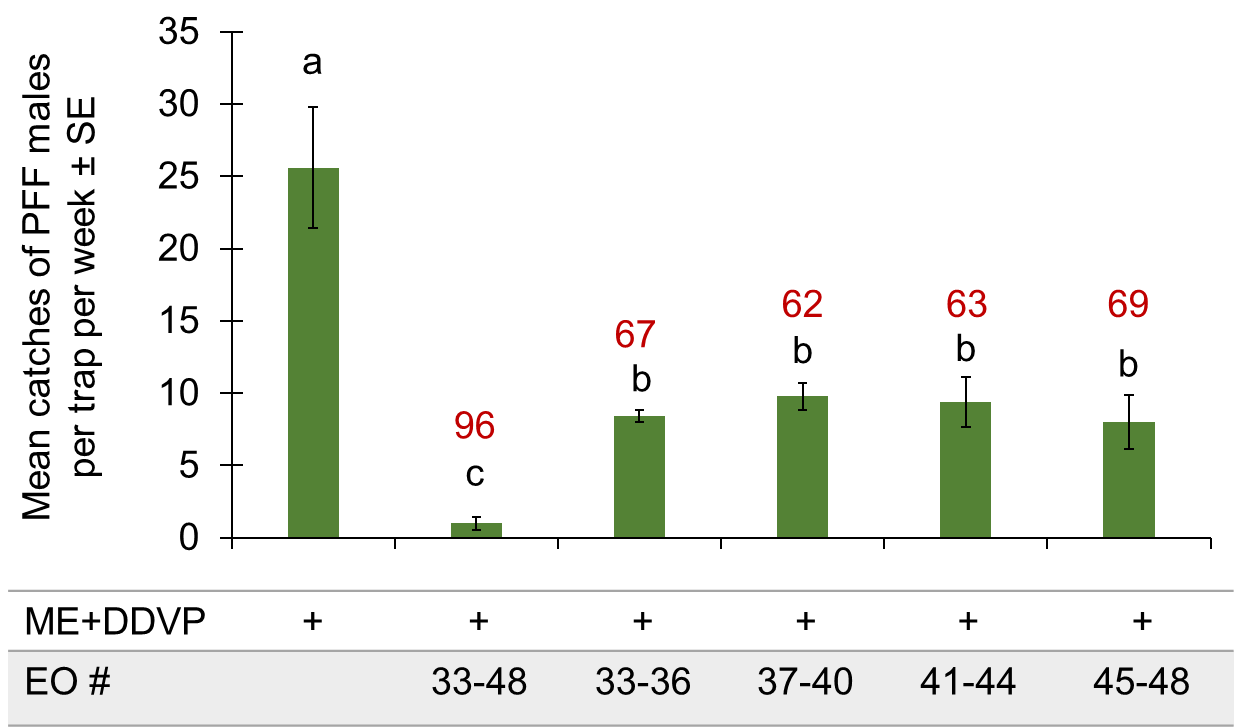


Fig. S4. Mean catches of PFF males by ME baits combined with EO #33-48 and sub-groups in mango orchard (7-14.3.2024) per trap per week ± SE (N=5 treatments per treatment). Bars with letters in common are not significantly different (GLM-Poisson regression with Tukey-Sidak post-hoc tests for multiple comparisons, α = 0.05). The number above each column (in red) represents the trap shutdown (%) for this bait and EO.

Table S1: Details of treatments in field trials

| **Orchard** | **Trap type, attractant, killing agent, replicate #** | **Treatments description** | **Dates from field display to counting** | **Experiment goal and title** |
| --- | --- | --- | --- | --- |
| Mango | Decis trap, ME (100 mg), DDVP, N=5 | 1. ME 2; ME + EOs #1-16; 3. ME + EOs #16-32; 4. ME + EOs # 33-48; 5. ME + EOs # 49-65; 6. ME + EOs # 66-82. Each EO (200 mg) dissolved in 300 mg paraffin oil. | 26.10.2023-1.11.2023 (I) | *Testing EO's #1-82* (Fig. 1) |
| Citrus | " | " | 6-13.11.2023 (II) |  |
| Mango | " | 1. ME; 2. ME + EOs #66-82; 3. ME + EOs #66-69; 4. ME + EOs #70-73; 5. ME + EOs #74-77; 6. ME + EOs #78-82. Each EO (200 mg) dissolved in 300 mg paraffin oil. | 15-22.11.2023 | *Testing EO's 66-82*  (Fig. 2) |
| Citrus | " | 1. ME; 2. ME + EOs #1-16; 3. ME + EOs #1-4; 4. ME + EOs #5-8; 5. ME + EOs #9-12; 6. ME + EOs #13-16. Each EO (200 mg) dissolved in 300 mg paraffin oil. | 28.11-5.12.2023 | *Testing EO's 1-16*  (Fig. S2) |
| " | " | 1. ME; 2. ME + EOs #17-32; 3. ME + EOs #17-20; 4. ME + EOs #21-24; 5. ME + EOs #25-28; 6. ME + EOs #29-32. Each EO (200 mg) dissolved in 300 mg paraffin oil. | 12-19.12.2023 | *Testing EO's 17-32*  (Fig. S3) |
| Mango | " | 1. ME; 2. ME + EOs #33-48; 3. ME + EOs #33-36; 3. ME + EOs #37-40; 4. ME + EOs #41-44; 5. ME + EOs #45-48. Each EO (200 mg) dissolved in 300 mg paraffin oil. | 7-14.3.2024 | *Testing EO's 33-48* (Fig. S4) |
| " | " | 1. ME; 2. ME + EOs #74-77, each EO (200 mg) dissolved in 300 mg paraffin oil; 3. ME + EO #74; 4. ME + EO #75; 5. ME + EO #76; 6. ME + EO #77. In each of the treatment # 3-6 there were four dispensers of the tested EO (200 mg) dissolved in 300 mg paraffin oil. Totally 800 mg of each EO in each trap. | 28.11-5.12.2023 | *Testing EOs sub-group 74-77* (Fig. 3) |
| " | Decis trap, ME (100 mg), DDVP, N=6 | 1. ME; 2. Four dispensers of 200 mg each of EO #75; 3. Four dispensers of 200 mg each of EO #76; 4. Two dispensers of 200 mg each of #75 plus two dispensers of 200 mg each of #76; 5. Eight dispensers of 200 mg each of #76. All EOs dissolved in paraffin oil 1:1.5 w/w. | 12-19.12.2023 (I) | *Testing EOs #75 (Yarrow) and #76 (Ylang-ylang)* (Fig. 4) |
| Citrus | " | 1. ME; 2. Four dispensers of 200 mg each of EO #75; 3. Four dispensers of 200 mg each of EO #76; 4. Two dispensers of 200 mg each of #75 plus two dispensers of 200 mg each of #76. All EOs dissolved in paraffin oil 1:1.5 w/w. | 26.12.2023-2.1.2024 (II) |  |
| Mango | " | 1. 25 mg EO in one PE sleeve of 1.25×1.25 cm^2^ (0.03125X); 2. 50 mg EO in one PE sleeve of 1.25×2.5 cm^2^ (0.0625X); 3. 200 mg EO in one PE sleeve of 5×2.5 cm^2^ (0.25X); 4. Four PE sleeves of 5×2.5 cm^2^ with 200 mg EO each (1X); 5. Sixteen 5×2.5 cm^2^ PE sleeves of 200 mg EO each (16X). Value 1X represents lures that were used in previous and next experiments that have the same specific release rate. All the other X values are related to increasing or decreasing surface area and the number of release points that affect the release rate of EO volatiles. | 28.12.23-4.1.2024 | *Repellent EO* #76 *(Ylang-ylang) dosage effect* (Fig. 5) |
| " | Decis trap, food bait (Ceratrap, 50 mL), DDVP, N=6 | 1. Food bait; 2. Food bait and two dispensers of 200 mg each of EOs #75 and #76 dissolved in PO 1:1.5 w/w; 3. DDVP dispenser alone. | 14-28.3.2024 (I) | *Testing the effect of EOs # 75+76 on the shutdown of female catches* (Fig. 6) |
| " | Decis trap, food bait (Ceratrap, 50 mL), DDVP, N=5 | 1. Food bait; 2. Food bait + two dispensers of 200 mg each of EOs #75 and #76; 3. Food bait with four dispensers of 200 mg each of EO #75; 4. Food bait with four dispensers of 200 mg each of EO #76; 5. DDVP dispenser alone. EOs dissolved in PO 1:1.5 w/w | 12-19.12.2024 (II) |  |
| " | " | 1. Food bait; 2. Food bait + two dispensers of 200 mg each of EOs #75 and #76; 3. Food bait with four dispensers of 200 mg each of EO #75; 4. Food bait with four dispensers of 200 mg each of EO #76. EOs dissolved in PO 1:1.5 w/w | 19.12.2024-8.1.2025 (III) |  |
| " | Decis trap, ME (100 mg), DDVP, N=6 | 1. ME; 2. Four dispensers containing 200 mg of Yarrow EO; 3. Four dispensers contained a mixture of 200 mg sabinene, 160 mg *β*-pinene, 40 mg eucalyptol, and 160 mg *β*-caryophyllene divided equally in four dispensers; 40 mg artemisia ketone, 120 mg germacrene D, and 80 mg chamazulene, each divided into four dispensers; 4. 800 mg of sabinene divided in four dispensers; 5. 800 mg of *β*-pinene divided in four dispensers; 6. 800 mg of eucalyptol divided in four dispensers; 7. 800 mg of *β*-caryophyllene divided in four dispensers; 8. DDVP alone.  The EO, sabinene, *β*-pinene, eucalyptol, *β*-caryophyllene and artemisia ketone were dissolved in paraffin oil 1:1.5 w/w and placed on a cotton roll in the PE sleeve. Germacrene D and chamazulene were placed neat in similar dispensers. | 28.10.2024-4.11.2024 (I) | *Testing EO #75 -Yarrow - and its major components* (Fig. 7) |
| " | " | 1. ME; 2. Four dispensers containing 200 mg of Yarrow EO; 3. Four dispensers that contained together a mixture of 200 mg sabinene, 160 mg *β*-pinene, 40 mg eucalyptol, and 160 mg *β*-caryophyllene plus four dispensers of each of 40 mg artemisia ketone, 120 mg germacrene D, and 80 mg chamazulene, each separately; 4. Four dispensers of 100 mg of artemisia ketone; 5. Four dispensers of 100 mg of germacrene D; 6. Four dispensers of 100 mg of chamazulene; 7. Four dispensers of 100 mg of *β*-caryophyllene; 8. DDVP alone.  EO, sabinene, *β*-pinene, eucalyptol, *β*-caryophyllene and artemisia ketone were dissolved in paraffin oil 1:1.5 w/w and placed on a cotton roll in the PE sleeve. Germacrene D and chamazulene placed neat. | 14-21.11.2024 (II) |  |
| " | " | 1. ME; 2. Four dispensers each containing 200 mg of Ylang-ylang EO; 3. Four dispensers with a mixture of 80 mg *β*-linalool, 80 mg geranyl acetate, 200 mg *β*-caryophyllene and 80 mg benzyl benzoates plus four dispensers with 120 mg germacrene D, four dispensers of a mixture of 80 mg *α*-caryophyllene, 40 mg *δ*-cadinene and 120 mg (*E,E*)-α-farnesene 4. Four dispensers of each 200 mg of *β*-linalool ; 5. Four dispensers each of 200 mg of geranyl acetate; 6. Four dispensers each of 200 mg of *β*-caryophyllene; 7. Four dispensers each of 200 mg of benzyl benzoate; 8. DDVP alone.  *β*-Linalool, geranyl acetate, *β*-caryophyllene and benzyl benzoate were dissolved in paraffin oil 1:1.5 w/w and placed on a cotton roll in the PE sleeve. Germacrene D, α-caryophyllene, *δ*-cadinene and (*E,E*)-α-farnesene were used neat. | 8-15.10.2024 (I) | *Testing EO #76 – Ylang-ylang - and its major components* (Fig. 8) |
| " | " | 1. ME; 2. Four dispensers each containing 200 mg of Ylang-ylang EO; 3. Four dispensers of 50 mg α-caryophyllene each; 4. Four dispensers of 50 mg (*E,E*)-α-farnesene each; 5. Four dispensers of 50 mg *δ*-cadinene.  EO was dissolved in paraffin oil 1:1.5 w/w and placed on a cotton roll in the PE sleeve. α-Cryophyllene, (*E,E*)-α-farnesene and *δ*-cadinene were used as neat. | 2-9.2.2025 (II) |  |
| " | Decis trap, food bait (Ceratrap, 50 mL), DDVP, N=6 | 1. Food bait; 2. Food bait plus four dispensers that each contained 200 mg of artemisia ketone impregnated neat on cotton disk in PE sealed sleeve; 3. DDVP dispenser alone | 16.1.2025-6.2.2025 | *Testing the effect of Artemisia ketone on the shutdown of female catches* (Fig.9) |
